# Retrospective study on the dengue fever outbreak in Puntland State, Somalia

**DOI:** 10.1186/s12879-024-09552-1

**Published:** 2024-07-25

**Authors:** Saaid Said Jama, Said Nuriye Abshir, Jibril Said Jama, Mohamed Mohamud Abdi

**Affiliations:** 1 Faculty of Medicine, University of Health Sciences, Bosaso, Puntland, Somalia; 2 Health emergencies program, World Health Organization, Garowe, Puntland Somalia; 3 Ministry of Health (Puntland), Alula, Puntland Somalia; 4 Ministry of Health (Puntland), Bosaso, Puntland Somalia

**Keywords:** Dengue fever, Dengue outbreak, Puntland, Somalia

## Abstract

**Background:**

Dengue infection is a mosquito-borne, endemic viral disease, particularly in developing countries. Here, we report the results of the clinicodemographic, serologic profile and the monthly occurrence of a recent dengue fever outbreak in Puntland State (Somalia).

**Methodology:**

We analyzed the data of 956 dengue-suspected patients who were investigated using the rapid diagnostic testing (RDT) method for detecting NS1 (dengue virus non-structural protein) and IgM antibodies employing the SD Biosensor Dengue Dou NS Ag and IgM test kit (Germany) at the Puntland Public Health Referral Laboratory from November 21, 2022, to May 27, 2023.

**Results:**

We found that 118 cases were positive for dengue among the suspected patients enrolled in the present study. Of these cases, 76.2% were dengue NSI positive, 13.6% were dengue IgM positive, and 10.2% were both NSI and IgM positive. The number of females and males in the confirmed cases was equal, and most (48.3%) were aged 20 years or less. 43.1% of them lived in the Nugal region, particularly in Garowe. Clinically, fever was the most frequent symptom (88.9%). The cases peaked in December 2022 but dropped from January to March, with a slight rise in February, and then increased in April and May 2023.

**Conclusion:**

This study highlights the clinicodemographic characteristics, seroprevalence, and monthly occurrence of dengue in Puntland. We recommend improving vector control measures, enhancing case management, strengthening dengue surveillance, developing an early warning system, and conducting future studies to characterize the circulating strains.

## Background

Dengue, one of the relevant public health issues, especially in tropical and subtropical countries, is a systemic febrile illness caused by the dengue virus (DENV), a single-stranded RNA virus that belongs to the genus Flavivirus and consists of four serotypes (DEN 1–4), transmitted to humans by the bite of the Aedes aegypti mosquito [1, 2]. The spread of the mosquito has been significantly aided by major demographic shifts, including population expansion, unplanned urbanization leading to poor housing, and the need for water storage [3].

Clinically, the illness can be asymptomatic or have manifestations such as fever, headache, retro-orbital pain, muscle and joint pain, rash, lymphadenopathy, hepatomegaly, nausea, and vomiting. In addition, some patients experience severe abdominal pain, persistent vomiting, tachypnea, hemorrhagic presentations, and neurological and mildly altered mental status features, which are considered to be warning signs [4–6].

Currently, serological tests used for diagnosing dengue infection rely on detecting either dengue non-structural protein 1 (NS1) antigen or dengue immunoglobulin M (IgM) through the enzyme-linked immunosorbent assay (ELISA) method [7]. Unfortunately, these tests might not be available in resource-limited settings. Therefore, less-sensitive, rapid diagnostic tests (RDTs) detecting dengue NS1 antigen or IgM are frequently used in these areas. Combining the two RDT tests raises their sensitivity for detecting dengue to 88.7% [8].

About 2.5 billion (> 40% of the world’s population) are at risk of dengue. According to World Health Organization (WHO) estimates, there may be 50–100 million dengue infections annually worldwide [9]. Epidemics of dengue have been reported in several African nations [10]. In Somalia, an outbreak of the dengue disease has been documented as early as 1985 in Hargeisa [11]. Also, infections of DENV-1, DENV-2, and DENV-3 and co-infections of DENV-1/2 and DENV-2/3 serotypes were identified in the Mogadishu outbreak in 2011 [12]. Recently, dengue was reported in the country in October 2022 [13]. In Puntland State (Somalia), the first case of dengue fever from the Sol region (Las Anod district) was confirmed on 21 November 2022. To our knowledge, no onset of dengue has been previously reported in the Puntland State. The present study, therefore, aims to describe the clinicodemographic, serologic profile, and monthly occurrence of a recent dengue fever outbreak in Puntland State (Somalia).

## Methods

### Study design

A descriptive observational, retrospective study was conducted using secondary data from the Puntland Public Health Referral Laboratory in the period from November 21, 2022, to May 27, 2023.

### Study area

This study was carried out in the Puntland State of Somalia, which is located in the northeastern part of the country and bordered by the Indian Ocean to its east and the Gulf of Aden to the north. The Puntland State consists of nine regions, namely, Bari, Karkar, Sanag, Nugal, Mudug, Sool, Ayn, Haylan, and Gardafue.

### Study population and sample size

All dengue-suspected cases tested and registered in the computerized record of the Puntland Public Health referral laboratory during the study period were included in the study. Patients with incomplete data on demographic, clinical, and serological profiles were excluded. A total of 956 dengue-suspected patients were included in this study.

### Diagnostic tool

All patients were tested for dengue rapid diagnostics of both NS1 and IgM using the SD Biosensor Dengue Dou NS Ag and IgM test kit (Germany) at the Puntland Public Health Lab. The SD Biosensor DENV NS1 has a sensitivity and specificity of 90.0%/90.2%, whereas the anti-DENV IgM has a sensitivity and specificity of 71.8%/83.5%.

### Data collection tool

Data were obtained from the laboratory in an Excel sheet. The data regarding demographic characteristics (sex, age, region, and district), clinical symptoms, testing dates, and results of dengue IgM and NS1 RDTs were collected.

### Statistical analysis

The data, obtained in a Microsoft Excel sheet, was checked for repetition. The data were complete regarding demographic characteristics, testing dates, and serologic results. Data were then imported into Statistical Package for the Social Sciences (SPSS) 20, and descriptive analysis was carried out by frequencies, and percentages and presented in tables and figures.

## Results

### Demographic characteristics of the dengue cases

Of the 956 dengue-suspected patients examined, we found that 118 were dengue-positive. The number of dengue-positive females and males was equal. Most of the dengue cases (48.3%) were aged 20 years or younger, and the age group (21–40 years) represented 42.4%, while the remaining 9.3% were 41 years of age or older (Table 1**).**

Regarding the geographic distribution of the dengue-positive cases, inhabitants of the Nugal region represented 43.1%, of which 38.1% were living in the Garowe district and 5% were in the Burtinle district. Residents of Mudug region (Galkayo district), Sool region (Las Anod district), and Karkar region (Gardo district) infected with dengue were 16.1%, 15.3%, and 13.6%, respectively, whereas 9.3% of the cases were from Bari region (Bossaso district). Finally, 2.5% of the cases were residents of the Sanag region (Dhahar district), as shown in Table 1.

**Table 1:** Demographic characteristics of dengue-positive cases (*n* = 118)

Characteristics	Frequency	Percentage (%)
Sex		
Female	59	50
Male	59	50
Age		
≤ 20 years	57	48.3
21–40 years	50	42.4
≥ 41 years	11	9.3
Region -District		
Bari – Bosaso district	11	9.3
Karkar – Gardo district	16	13.6
Mudug – Galkayo district	19	16.1
Nugal Garowe district Burtinle district	51 45 6	43.1 38.1 5.1
Sool- Las Anod district	18	15.3
Sanag – Dhahar district	3	2.5

### Clinical profile of dengue cases

Furthermore, the clinical symptoms observed in the dengue-infected patients and their frequencies after we excluded 10 cases with missing clinical symptoms are summarized in Table 2.

**Table 2:** Clinical symptoms of dengue-positive cases (*n* = 108)

Clinical features	Frequency	Percentage (%)
Fever	96	88.9
Myalgia	38	35.2
Rash	29	26.9
Gastrointestinal symptoms Nausea and vomiting Abdominal pain and diarrhea	27 5	25 4.6
Arthralgia	20	18.5
Headache	16	14.8
Neurologic symptoms (confusion, convulsion, vertigo, photophobia)	7	6.5
Respiratory symptoms (sore throat, cough, chest pain)	5	4.6
Fatigue	5	4.6

### Severity of the dengue patients

In this study, only 7 patients aged 18 years or less were hospitalized; of them, 4 were females and 3 were males (Table 3).

**Table 3:** Severity of the dengue cases (*n* = 108)

Item	Frequency
Outpatient	101
Hospitalization Age (≤ 18 years) Sex Females Males	7 7 4 3

### Serology profile of the dengue-positive cases

Finally, as reported in Table 4, out of the 956 dengue-suspected patients examined in the present study, 118 cases were confirmed dengue-positive. The current study showed that 90 (76.2%) of the dengue cases were dengue NSI positive, 16 (13.6%) of them were dengue IgM positive, and the remaining 12 (10.2%) were both dengue IgM and NSI positive **(**Table 4).

**Table 4:** Serologic profile of dengue cases

Serology result	Frequency	Percentage (%)
Total cases	956	
Dengue Positive cases	118	12.3
Dengue NSI Only +	90	76.2
Dengue IgM only +	16	13.6
Both Dengue IgM and NSI +	12	10.2

### Monthly distribution of dengue cases

**Fig. 1:**
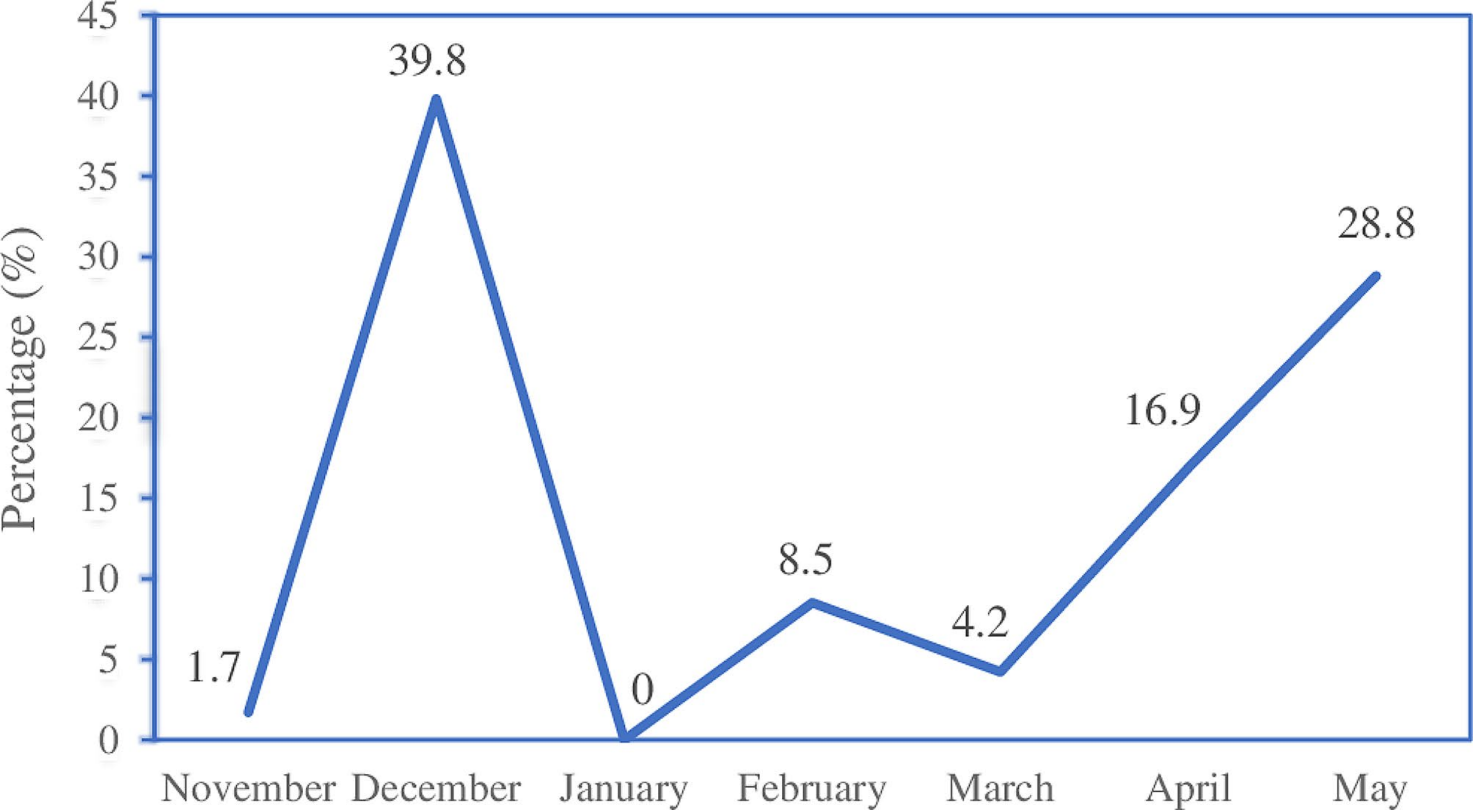
Monthly occurrence of dengue cases

The frequency of occurrence of the dengue cases varied during the study period. The monthly distribution of the dengue cases in Puntland is reported in Fig. 1.

## Discussion

Currently, dengue infection is endemic in many parts of the world [14]. Patients with this infection may present with various clinical features that resemble other acute febrile illnesses, making its diagnosis difficult [15]. Therefore, efficient clinical and laboratory evaluations are cornerstones of care for dengue-suspected patients.

Of the 956 dengue-suspected patients examined in this study, we found 118 dengue positive cases. The number of males and females infected was equal, contrasting the results of earlier reports, where female preponderance was observed [16–19]. This observation also contrasts with other studies that found a higher rate of dengue infection among males [20–22]. Further, we found that most (48.3%) of the infected cases were 20 years of age or less. This result is comparable to that (52%) reported in Darfur, Sudan [23]. The higher vulnerability of this age group to the disease might be due to their exposure to mosquito bites in schools, during outdoor activities, and indoor biting since many of them stay home longer. Also, the clinical features are known to be more apparent in children [24]. In the present study, we investigated the distribution of the disease in all regions of Puntland State (Somalia) (see Table 1). Most of the dengue-positive cases were in the Nugal region, particularly in Garowe, the capital city of Puntland. This city has experienced rapid population growth, fast urbanization, an increase in internally displaced persons (IDPs) camps, and the influx of large returnees and migrants [25]. The higher prevalence of dengue in Garowe could be related to these demographic factors and climate changes.

Clinically, dengue patients frequently present with a triad of fever, pain, and rash. Nevertheless, gastrointestinal and bleeding symptoms might occur in variable proportions [26]. Our study showed that fever was the most common clinical symptom (88.9%), which is consistent with earlier studies [27, 28]. The second most frequent symptom (35.2%) was myalgia (Table 1). A higher frequency of myalgia among dengue patients was described before [5, 29, 30]. This was followed by skin rash and gastrointestinal symptoms, consistent with a study conducted in Saudi Arabia [31].

In our study, most of the dengue-positive cases were mild and treated as outpatients. Only seven pediatric cases required hospital admission. The increased risk of severe forms of dengue resulting in hospitalization and mortality among children in tropical areas has been documented [32]. Moreover, in pediatric patients, dengue frequently causes profound vascular leakage and abrupt shock [33].

In this study, the overall seroprevalence of dengue was found to be 118 (12.3%), which is lower than the results of other studies carried out in Kenya (61.2%) and Sudan (42%) [34, 35]. The study also showed that 13.6% of dengue cases were dengue IgM antibody positive, which is in line with the 14.7% reported from Eastern Italy [36]. This result was lower than the 21% reported in northwest Ethiopia [37]. Furthermore, the present study revealed a higher positive rate (76.2%) of dengue NS1 among the study participants (Table 3). It is known that NS1 antibody is useful in the early stages of the disease, especially in the days 3–5 after the onset of the illness, when viremia levels might be undetectable and anti-IgM antibody levels have yet to rise [38]. Thus, the higher positive rates of NS1 in this study could be attributed to patients presenting in the acute phase of the illness. The discrepancies in the seroprevalence of dengue might be explained by the differences in environmental conditions, the sample sizes, and the diagnostic methods employed.

Rainfall and temperature play an important role in mosquito proliferation and the incidence of dengue illness [39]. The temperature in Puntland varies from 27 °C to 37 °C, and the spring season (April–June) is regarded as the principal rainy season, while the autumn season (October–December) is a short rainy season [40]. The seasonal distribution of dengue throughout the study period is reported in Fig. 1. Dengue cases peaked in December 2022, fell from January to March with a slight rise in February, and then increased in April and May 2023. The monthly variation in dengue cases could be due to weather changes and rainfall. A previous study revealed the impact of climate variability on the occurrence of dengue [29].

Although this study presents evidence of the dengue fever outbreak in Puntland State, Somalia, some limitations should be considered. Firstly, the study used secondary data subjected to incomplete or missing information. As discussed earlier, there were cases with missing clinical features. Secondly, we were unable to characterize the virus since we used serologic tests for the diagnosis. Thirdly, we were unable to identify the source of dengue infection or provide detailed information related to the disease transmission. Lastly, data on dengue case outcomes was not recorded, so assessing the patients’ outcomes was impossible.

## Conclusion

In conclusion, we found that of the 956 dengue-suspected patients investigated, 118 cases were dengue-positive. Patients aged 20 years or younger were the most infected. Fever was the most frequent clinical symptom in the patients. Cases were highest in December 2022, followed by May 2023. We recommend improving vector control measures, enhancing case management, strengthening dengue surveillance, developing an early warning system, and conducting future studies to characterize the circulating strains.

## Data Availability

The datasets used and/or analyzed during the current study available from the corresponding author on reasonable request.

## Abbreviations

RDTs: Rapid diagnostic tests
DENV: Dengue virus
RNA: Ribonucleic acid
NS1: Non-structural protein 1 antigen
IgM: Immunoglobulin M
ELISA: Enzyme-linked immunosorbent assay
WHO: World Health Organization
IDPs: Internally displaced persons
