# Correction: Retrospective study on the dengue fever outbreak in Puntland State, Somalia

**DOI:** 10.1186/s12879-024-09692-4

**Published:** 2024-08-02

**Authors:** Saaid Said Jama, Said Nuriye Abshir, Jibril Said Jama, Mohamed Mohamud Abdi

**Affiliations:** 1 Faculty of Medicine, University of Health Sciences, Bosaso, Puntland, Somalia; 2 Health emergencies program, World Health Organization, Garowe, Puntland, Somalia; 3 Ministry of Health (Puntland), Alula, Puntland, Somalia; 4 Ministry of Health (Puntland), Bosaso, Puntland, Somalia

**Correction: Jama BMC Infectious Diseases**

10.1186/s12879-024-09552-1

Following publication of the original article [1], we have been notified that the affiliation numbers in the authorship line ware incorrect.

Originally published: Saaid Said Jama1,2,3,4*, Said Nuriye Abshir1,2,3,4, Jibril Said Jama4 and Mohamed Mohamud Abdi3

Correct: Saaid Said Jama1*, Said Nuriye Abshir2, Jibril Said Jama3 and Mohamed Mohamud Abdi4

The original article has been corrected.
